# Imaging immunometabolism *in situ* in live animals

**DOI:** 10.1097/IN9.0000000000000044

**Published:** 2024-07-31

**Authors:** Nicole Molnar, Veronika Miskolci

**Affiliations:** 1 Department of Microbiology, Biochemistry and Molecular Genetics, New Jersey Medical School, Rutgers Health, Rutgers University, Newark, NJ, USA; 2 Center for Cell Signaling, Rutgers Health, Rutgers University, Newark, NJ, USA; 3 Center for Immunity and Inflammation, Rutgers Health, Rutgers University, Newark, NJ, USA

**Keywords:** immune cells, spatial metabolomics, enzymatic activity assay, Raman, FLIM, lifetime imaging, NAD(P)H, FAD, optical redox ratio

## Abstract

Immunometabolism is a rapidly developing field that holds great promise for diagnostic and therapeutic benefits to human diseases. The field has emerged based on seminal findings from in vitro and ex vivo studies that established the fundamental role of metabolism in immune cell effector functions. Currently, the field is acknowledging the necessity of investigating cellular metabolism within the natural context of biological processes. Examining cells in their native microenvironment is essential not only to reveal cell-intrinsic mechanisms but also to understand how cross-talk between neighboring cells regulates metabolism at the tissue level in a local niche. This necessity is driving innovation and advancement in multiple imaging-based technologies to enable analysis of dynamic intracellular metabolism at the single-cell level, with spatial and temporal resolution. In this review, we tally the currently available imaging-based technologies and explore the emerging methods of Raman and autofluorescence lifetime imaging microscopy, which hold significant potential and offer broad applications in the field of immunometabolism.

## 1. Introduction

Flow cytometry, seahorse assay, metabolomics, and flux analysis by isotope tracing have been the workhorse methods in immunometabolism ^[1-4]^. These methods are well-suited for in vitro studies in immunometabolism; however, except for flow cytometry, they provide bulk measurements that lose information about the heterogeneity in a sample. Measurements in vitro also have limitations; they fail to capture the complex tissue microenvironment in which immune cells perform their functions. Immune cells do not exist in isolation; they interact and engage in cross-talk with neighboring cells. In addition, they are exposed to a dynamic mixture of extracellular signals that influence their phenotype and behavior. Furthermore, the nutritional needs of immune cells may not be reflected in vitro ^[4,5]^. Traditional cell culture media are poorly formulated and fail to model the metabolite composition of human plasma ^[6,7]^. In addition, a recent study brought attention to the issues of limited oxygen availability in standard cell culture ^[8]^. The suboptimal fidelity of standard cell culture conditions in reflecting in vivo conditions directly impacts metabolic assessments using in vitro models. Another consideration is that immune cells are not only heterogeneous but also have a plastic nature and readily respond to their new environment. This led to concerns that removing immune cells from their native microenvironment for ex vivo analysis alone may alter their metabolic profiles ^[4]^. These considerations have been fueling a shift from bulk to single-cell analysis, and from in vitro to in vivo, or more accurately, in situ analysis. This shift in the field has driven the use of single-cell approaches such as single-cell and spatial transcriptomics and metabolomics ^[4]^, and SCENITH ^[9]^. However, these methods still lack other important analytical capabilities, such as temporal resolution and in vivo measurements. Imaging-based approaches can address all these analytical needs, including single-cell measurements, spatiotemporal resolution, and compatibility with living organisms.

Not surprisingly, spatial metabolomics and metabolomics in combination with microscopy, have been developing for years ^[10-12]^. In the recent publication of SpaceM, researchers have demonstrated that matrix-assisted laser desorption ionization (MALDI) can be combined with light microscopy, named MALDI-imaging mass spectroscopy (MS), to generate a metabolome map of a single cell ^[13]^. SpaceM can detect >100 metabolites and lipids from >1000 cells per hour ^[13]^; it can also detect drugs and proteins. This broad range in detection makes MALDI-imaging MS an undeniably powerful in situ method. Another developing technology in spatial metabolomics is spatial isotope tracing, to enable flux analysis with spatial resolution ^[14,15]^. These emerging methods in spatial metabolomics will have a profound impact on our understanding of the metabolic regulation of immune cell function.

A metabolic imaging method that has gained popularity since its publication is enzyme histochemistry ^[16]^. This approach analyzed tissue sections with traditional immune cell staining in combination with dehydrogenase activity measurements. The authors successfully measured five different enzymes, including glucose-6-phosphate dehydrogenase (G6PD) in the pentose phosphate pathway (PPP), glyceraldehyde 3-phosphate dehydrogenase (GAPDH) in glycolysis, lactate dehydrogenase (LDH) in lactate fermentation, and isocitrate dehydrogenase (IDH) and succinate dehydrogenase (SDH) in the tricarboxylic acid (TCA) cycle, to evaluate the metabolic profile of single cells in intact tissues from healthy and tumor colon tissues ^[16]^. Multiple studies have employed this or a similar approach ^[17-19]^, demonstrating the usefulness and ease of implementation of enzyme histochemistry.

Although spatial metabolomics and enzyme histochemistry are undeniably powerful tools due to their broad coverage, they require the use of fixed samples and extensive sample preparation and have limited temporal resolution. Imaging approaches that allow for using living tissues and intact animals provide the opportunity for true in situ assessment. Live imaging approaches best preserve a local microenvironment in its authentic state and can provide subcellular spatial resolution and temporal resolution on the scale of seconds. These considerations motivated the development of fluorescent probes and fluorescence-based biosensors, which have been used for decades for studying intracellular signaling in living cells and tissues to gain insight into the dynamic localization, abundance, and activity of the molecule of interest ^[20]^. The main components of a genetically encoded fluorescent biosensor are fluorescent protein(s) and a sensing domain. The binding of the target molecule causes a conformational change in the sensor, thereby modulating the fluorescence signal, which can be measured by fluorescence microscopy. Critical considerations for a successful biosensor include intracellular expression levels, specificity, and dynamic range. Biosensors can be easily modified to direct expression into subcellular compartments, such as the mitochondria, allowing compartment-specific metabolite analysis. We direct the reader to several comprehensive reviews on this topic that include discussions of the limitations of genetically encoded biosensors and extensive lists of biosensors available for metabolic analysis ^[21-27]^. Here, we provide a partial list of potentially useful sensors in Table 1. Below, we discuss Raman microscopy and autofluorescence lifetime imaging microscopy, which have the advantage of being readily applicable to human samples as they are already in use in the clinic ^[75-77]^ due to their label-free nature. Although these methods are extensively discussed in the literature, few reviews have focused on their applications in immunological studies in living animals, as such reports are limited and still emerging. Here, we review their potential use in metabolic imaging of immune cells in living, whole animals and highlight recent studies with successful applications.

## 2. Raman microscopy

Raman spectro-microscopy techniques have garnered considerable attention across scientific domains due to their versatility in chemical sensing, biological imaging, and component characterization ^[78]^. Operating on the principles of the Raman effect, these technologies can discern molecular composition by analyzing the frequency and intensity of Raman-scattered light, offering insights into the physiochemical makeup of a sample ^[75,76]^. The Raman effect describes the inelastic scattering of photons by matter, induced by molecular vibration and energy transfer between incident photons within the molecule ^[75]^. The transfer of energy causes a shift in the wavelength of scattered light that is highly specific to the physiochemical properties of the sample, such that its composition, conformational state, and polarization can be determined ^[76]^. Therefore, Raman spectroscopy offers a label-free and non-destructive technique that relies on the interaction of light with chemical bonds within molecules, where incident light from a laser source is scattered at specific wavelengths to discern the structure of the molecule ^[78]^. Raman spectra are sensitive to differences in several groups of biomolecules, including amino acids, nucleic acids, lipids, carbohydrates, metabolites, and resonant molecules, and more complex biological materials such as fungal spores and bacteria ^[79]^. The use of Raman spectroscopy applications in immunological studies has increased in recent years ^[80]^. For example, Raman spectral data can be leveraged in conjunction with principal component analysis (PCA), enabling the differentiation of macrophage activation into M1 and M2 states ^[81,82]^. Although these studies highlight the utility of Raman spectroscopy in distinguishing between immune cell phenotypes, the in vitro nature of these experiments has their caveats, as the plastic nature of macrophages may cause phenotypic alterations when removed from their native environment. Moreover, solely relying on spectroscopic techniques neglects spatial and temporal information, which poses a limitation, particularly in studies of immunometabolism where context is paramount. To address these limitations, Raman micro-spectroscopic imaging techniques, such as confocal Raman micro-spectroscopy (CRM), coherent Raman imaging (CRS) techniques, and broadband coherent anti-stokes Raman scattering (BCARS) microscopy, have been employed to analyze the biochemical changes within (immune) cells and their local microenvironment ^[76]^.

By integrating Raman scattering properties with confocal laser scanning microscopy, CRM techniques have emerged, facilitating label-free imaging based on molecular vibrations ^[83]^. This spontaneous or “non-coherent” Raman imaging approach offers spatial resolution of chemical components and internal structures, demonstrating promise for in vitro and ex vivo analysis of immune cells. For instance, researchers employed CRM to characterize immune cells according to their biochemical composition ^[84]^. They captured Raman spectral images of single immune cells using a confocal Raman microscope. Based on their spectral profile, they could differentiate between fixed T and B lymphocytes. T cells were identified by the presence of β-carotene in the cytoplasm, while B cells exhibited higher contributions of nucleic acids in their spectra ^[84]^. The results of this study validated those of a 2016 study, where researchers sought to discriminate between live T and B cells using Raman spectro-microscopic techniques ^[85]^. Using a coherent laser for the excitation, collecting the backscattered light by the objective lens, and converting the spectral information onto a CCD detector, not only were they able to differentiate between T and B lymphocytes, but they were also able to decipher between naive and active T cells based on the differences in their Raman spectral images ^[85]^. For example, by analyzing live cells in vitro, scientists successfully observed the progressive transition of naive T cells into activated states, noting that Raman images of activated T cells showed a greater abundance of cytochrome C, indicating higher metabolic activity when compared to naïve T cells. The results of this study highlight the efficacy of Raman spectral data in discerning dynamic changes in the intracellular metabolism within individual cells in a heterogeneous population ^[85]^. Although T cell differentiation has been relatively well characterized with Raman microspectroscopic techniques ^[85,86]^, these studies only scratch the surface of the utility of these tools in immune cell discrimination. Validating and expanding on these findings, another study investigated the feasibility of using CRM to discriminate between activated and non-activated lymphocytes (T and B) and macrophages (monocytes) using in vitro and ex vivo models ^[87]^. In the in vitro studies, cell lines and primary cells (monocytes and lymphocytes) were stimulated with various agents. The activation responses were confirmed using flow cytometry or ELISA. For the ex vivo model, mixed peripheral blood mononuclear cells (PBMCs) were isolated from healthy donors and subjected to similar activation protocols. Similarly to the above-described experiments, researchers identified specific biomolecular markers or signatures associated with immune cell subtypes in their resting and activated states by analyzing the Raman spectra of these differentially activated immune cell types ^[87]^. For monocytes, spectral features linked to proteins, nucleic acids, and lipids were significant, reflecting changes in cellular components during activation. For lymphocytes, markers included variations in nucleic acid and protein content, corresponding to the biochemical changes upon activation. These spectral signatures enabled the distinction between resting and activated states of immune cells, facilitating non-invasive monitoring of immune responses. Not only is CRM useful in the identification of cell types based on their inherent characteristics, but it also proves useful in differentiating between ‘normal’ and metabolite-deficient cells. For example, one study used CRM to resolve fumarate spatially, an intermediate metabolite in the TCA cycle, within living murine kidney cells in vitro and tissues ex vivo ^[88]^. This work demonstrates the capability of CRM in distinguishing between cells with normal and deficient levels of Fumarate hydratase (Fh1), an enzyme involved in fumarate metabolism, based on fumarate concentration. Additionally, CRM revealed the intracellular distribution of fumarate, with the highest concentration observed in the mitochondria of Fh1-deficient cells. Furthermore, CRM accurately identified Fh1 loss in kidney tissues from a mouse model. By understanding the unique spectral features of fumarate, CRM could resolve variability in the distribution and concentration of the metabolite at subcellular and tissue levels, providing valuable insights into its cellular localization and metabolic activity. While the focus of the study may not be on immune cells, its findings hold significant implications for the field of immunometabolism. The methodology employed in this study, specifically the identification and subcellular localization of distinct metabolic intermediates using Raman micro-spectroscopy, can be applied to investigate immune cell metabolism. This approach allows for detailed profiling of cellular metabolism, enabling the study of metabolic pathways and their changes during immune cell activation or in response to different stimuli. Taken together, these studies demonstrate the feasibility of CRM in discriminating immune cell type and activation state, offering valuable insights into the molecular mechanisms underlying immune responses and metabolic rewiring. However, the ex vivo nature of these experiments poses a subset of challenges in the study of immunometabolism due to the plasticity of immune cells.

Expanding on the imaging capabilities of CRM, high-resolution confocal Raman spectroscopic imaging (cRSI) is an alternative method for in vivo imaging and biomolecular analysis of living organisms. The applicability of cRSI was demonstrated by imaging immune responses in zebrafish embryos, showcasing its potential for characterizing biomolecular information and probing local biochemical variations ^[89]^. The study conducted time-lapse imaging of immune responses in live zebrafish embryos, revealing cellular changes associated with wound responses. The methodology showcases promising applications for live imaging in a living animal. However, it did not attempt to identify differentially activated cells or resolve biomolecular composition at the single-cell level.

The functional limitations of confocal Raman spectro-microscopic techniques pose challenges in resolving cell heterogeneity and subcellular structures in live samples due to long acquisition times per pixel ^[90]^. Consequently, noncoherent Raman techniques are not ideal for real-time imaging of intracellular molecular distributions in vivo. However, recent advancements in coherent Raman imaging (CRS) techniques, including stimulated Raman spectroscopy (SRS) and coherent anti-Stokes Raman scattering (CARS), potentially offer enhanced capabilities for in vivo applications by significantly reducing the time required for Raman signal acquisition ^[91]^. For example, using a backward multiplex CARS spectroscopic imaging system, researchers could visualize and spectroscopically identify intracellular organelles in vitro, even with low exposure time. These nonlinear and coherent techniques utilize multiple laser beams to generate more robust Raman spectra capable of penetrating deeper into tissues, therefore boosting the spatial resolution of previously diffraction-limited (non-coherent) spectroscopic imaging ^[92]^. A comprehensive review of CRS microscopy techniques highlighted their utility in live cell imaging for deciphering single-cell metabolism based on various biomolecules such as lipids, proteins, nucleic acids, and glucose ^[93]^. The review mainly focuses on cancer cell metabolism as these CRS methods have yet to be applied in immunometabolism research. However, it highlights the potential these single-cell techniques have in imaging and discriminating between differentially activated immune cell types and states in vivo.

Recent advances in single-cell Raman spectroscopy further elucidate the potential of single-cell metabolic profiling in (immune) cells. For instance, stimulated Raman scattering (SRS) microscopy was combined with deuterium oxide (D_2_O) probing for live imaging of metabolic dynamics via their pioneered technique of DO-SRS microscopy ^[94]^. By introducing D_2_O to the biological systems under investigation, deuterium can form O-D, S-D, and N-D bonds, or C-D bonds via reversible non-enzymatic hydrogen/deuterium exchange, or irreversible enzymatic exchange, respectively ^[94]^. Deuterium atoms are assimilated into biomolecules, including but not limited to lipids, proteins, and nucleic acids, via metabolic pathways ^[94]^. This incorporation of deuterium serves as a stable isotope tracer, allowing for the monitoring of metabolic fluxes. In this novel technique, deuterium-labeled metabolites act as endogenous contrast agents for SRS imaging. By tuning the excitation wavelengths to target Raman shifts corresponding to deuterium-containing molecular vibrations, such as C-D or O-D bonds, specific metabolic pathways or compartments can be visualized and quantified. DO-SRS microscopy methods enabled real-time imaging of metabolic processes, including lipid and protein metabolism within living animal models, such as in *C. elegans* and zebrafish, as well as in mouse tissues. Using *C. elegans* as a model, researchers explored the dynamics of lipogenesis and lipid degradation under different fasting and feeding conditions, and visualized protein and lipid metabolism during germline development. By incorporating deuterium from D_2_O into lipids, they could map the distribution and synthesis of these macromolecules in the nematodes. Dynamic imaging, coupled with SRS microscopy allowed for tracking of real-time metabolic changes in response to environmental or genetic modifications, providing insights into how these metabolic pathways are regulated to utilize lipids during different life stages or stress conditions. Expanding on the capability of DO-SRS in in vivo studies, researchers used DO-SRS in conjunction with fluorescent microscopy to track the metabolic activity of specific cell lineages during zebrafish embryogenesis. This co-labeling enabled the tracking of time-dependent metabolic activity of specified cell types throughout development. Small, transparent model organisms, such as *C. elegans* and zebrafish, are well suited for in vivo metabolic imaging using DO-SRS; accessing deeper tissues, such as in murine organs, is still a challenge. However, with technological advancement, it may eventually be possible to use devices similar to coherent Raman scattering endoscopes to study metabolism through optical biopsies. Overall, this investigation underscored the versatility, sensitivity, precision, and non-invasive nature of DO-SRS in live imaging, offering novel insights into the metabolic dynamics governing biological processes.

Broadband coherent anti-stokes Raman scattering (BCARS) microscopy, another suitable method for live imaging, was explored in correlating Raman spectral changes with gene expression profiles in the *C. elegans* hermaphrodite gonad ^[95]^. Raman micro-spectroscopy holds the potential for distinguishing cell phenotypes by their metabolic profiles, which are associated with transcriptomic activity. However, linking these spectral alterations to distinct signaling pathways necessitates precise control over experimental conditions and rapid spectral acquisition on a large scale. Using BCARS microscopy, this investigation showcased that spatio-spectral patterns align with gene expression profiles, implying BCARS is a promising proxy for spatially resolved -omics analyses ^[95]^. Although these coherent techniques overcome many of the barriers associated with live-image acquisition through spontaneous Raman scattering, imaging in deeper tissues remains an accessibility issue. Overall, these novel Raman-based techniques offer significant advancements in in vivo imaging, providing enhanced spatial and temporal resolution compared to traditional Raman processes. Therefore, these techniques hold great promise for characterizing immune cell metabolism and dynamics, offering valuable insights into immune responses and cellular functions.

By leveraging the unique properties of Raman scattering to probe molecular composition with high specificity and sensitivity, these techniques enable the characterization of immune cell types and states based on their biochemical signatures. Raman spectro-microscopy techniques have revolutionized our ability to analyze immune cell metabolism and dynamics, offering unprecedented insights into cellular functions and responses. From discriminating between different cell types and activation states to tracking metabolic processes in real time, these techniques have proven invaluable in both in vitro and ex vivo settings. However, challenges such as the motile nature of immune cells and limitations in spatial and temporal resolution persist, particularly in live imaging scenarios. Emerging technologies, such as coherent Raman imaging, show promise in overcoming these barriers, paving the way for more comprehensive studies of immunometabolism in vivo. As these methodologies continue to evolve, they hold the potential to uncover new mechanisms underlying immune responses and metabolic pathways, thereby advancing our understanding of immune function and dysfunction in health and disease.

## 3. Fluorescence lifetime imaging microscopy (FLIM) of NAD(P)H and FAD

**Figure 1.**
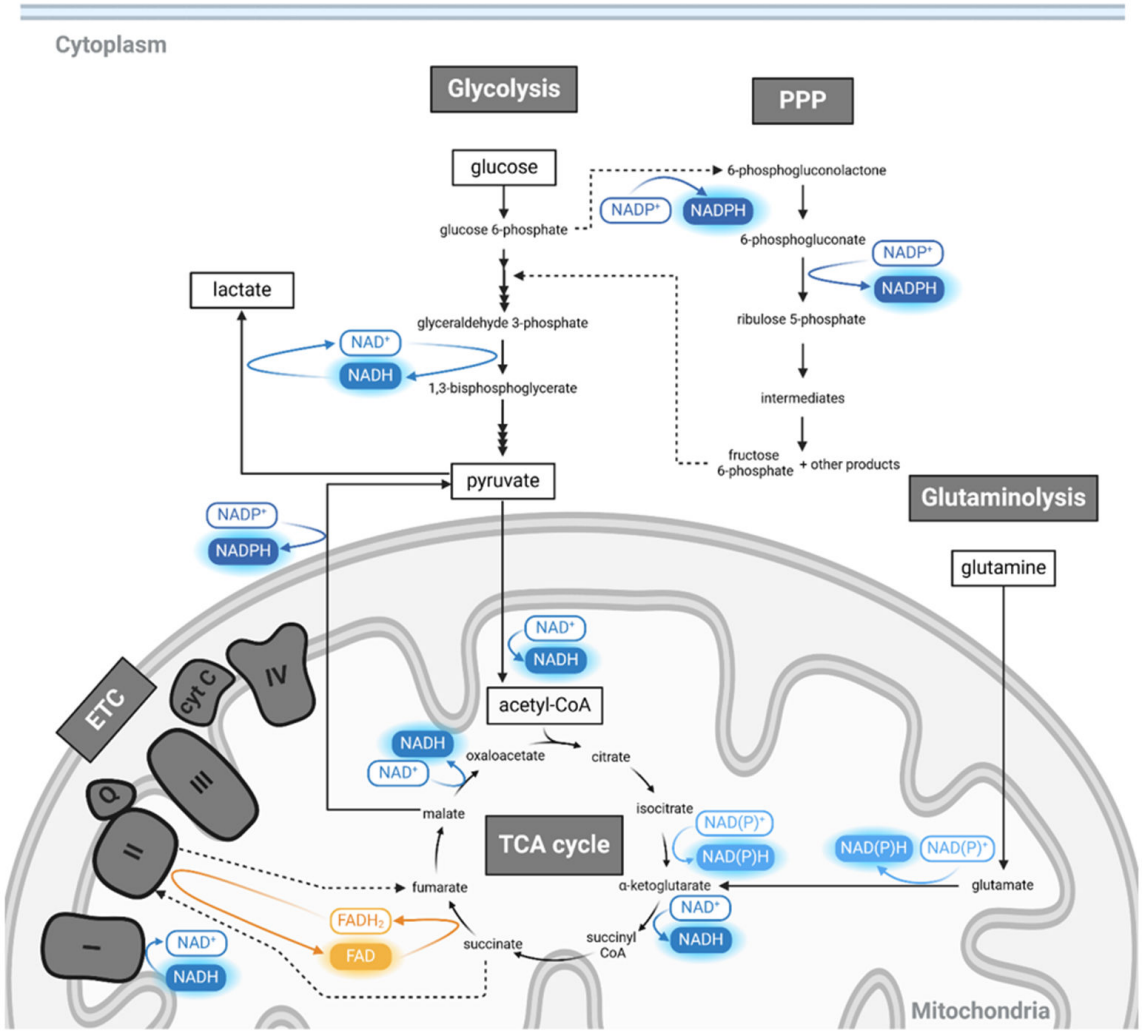
Simplified overview of cellular metabolic pathways that utilize or generate NAD(P)H and FAD. Pathways represented, including glycolysis, the pentose phosphate pathway (PPP), electron transport chain (ETC), the tricarboxylic acid (TCA) cycle, and glutaminolysis are boxed in gray. Key metabolites (glucose, lactate, pyruvate, glutamine, and acetyl CoA) are highlighted within boxed outlines. NADH, NADPH, and NAD(P)H have the same blue glow to represent their overlapping autofluorescence spectra but are filled with different shades to discriminate their roles in metabolism visually. FAD is shown in orange. This figure was created with BioRender.com.

Nicotinamide adenine dinucleotide (NADH) and flavin adenine dinucleotide (FAD) are endogenous coenzymes that serve as electron carriers during cellular respiration to produce energy in the form of adenosine triphosphate (ATP) (Figure 1). Given their critical roles in cellular metabolism, they have been used to monitor intracellular metabolism ^[96,97]^. NADH and FAD exist in two forms, oxidized NAD^+^ and FAD, and reduced NADH and FADH_2_, where the reduced NADH and oxidized FAD exhibit autofluorescence. NADH has optimal excitation at 350 nm (750 nm with two-photon excitation) and maximal emission at 440-470 nm, while FAD is excited at 450 nm (890 nm with two-photon excitation) with maximal emission at 515–535 nm ^[96-98]^. The one-photon excitation of NADH in the UV range poses a concern for phototoxicity for live imaging of cells or tissues. However, this can be circumvented by employing two-photon microscopy ^[97]^. The separable maximal emission spectra of NADH and FAD enable the simultaneous measurement of both coenzymes to quantify dynamic changes in cellular metabolism. Early work by Britton Chance and colleagues pioneered the use of NADH and FAD fluorescence intensity to quantify the optical redox ratio (I_FAD_/I_NADH_) that is still widely used today ^[96-100]^. The optical redox ratio provides an assessment of the global intracellular metabolic activity based on the oxidation-reduction state of the cell. The intracellular balance of NADH and FAD will dictate the metabolic state of the cell and will be reflected by relative changes in the optical redox ratio. For example, an increase in glycolysis is predicted to cause an increase in NADH and a decrease in FAD intensities, which would cause a decrease in the optical redox ratio. Note that there are several versions of the optical redox ratio ^[99]^, and the change in optical redox ratio will depend on the specific definition in use, which the authors typically provide.

The fluorescence lifetime, which measures the time a molecule spends in the excited state before decaying back to the ground state, of NADH and FAD has been recently explored in metabolic imaging ^[98,101]^. Fluorescence lifetime is sensitive to various factors in its molecular microenvironment, such as pH, viscosity, and temperature. Lifetime-based measurements offer several advantages over intensity-based measurements, such as independence from fluorophore concentration ^[98]^. NADH and FAD exist in free, unbound and protein-bound forms, and the changes in lifetime have been shown to depend on the enzyme-binding activity of the coenzymes. NADH in a free, unbound state has a shorter lifetime than a protein-bound state due to quenching by the adenine moiety of the molecule. FAD behaves differently, where unbound FAD has a long lifetime, and protein-bound has a short lifetime. A double-exponential decay model is used to estimate the fluorescence decay of the coenzymes to account for the short and long components. The mean lifetime (τ_m_) can be calculated as the amplitude-weighted average of the short and long lifetime components, τ_m_ = α_1_τ_1_ + α_2_τ_2_, where τ_1_ is the short lifetime for unbound NADH and protein-bound FAD, τ_2_ is the long lifetime of protein-bound NADH and unbound FAD, and α_1_ and α_2_ represent relative contributions from unbound and protein-bound NADH, respectively, and the converse for FAD ^[99]^. Since the fluorescence lifetimes of unbound and protein-bound coenzymes are distinguishable, these measurements can provide additional biological information and be used as metabolic indicators. For example, an increase in glycolysis is predicted to cause an increase in unbound NADH (τ_1_) and a decrease in NADH τ_m_, while an increase in unbound FAD (τ_2_) and an increase in FAD τ_m_^[99]^. In addition to the optical redox ratio and mean lifetime, the lifetime components can be applied in other indicators to evaluate cellular metabolism at a greater depth, such as the α_1_/α_2_ ratio, the fluorescence-lifetime redox ratio (FLIRR), the Mitochondrial-Cytoplasmic Ratio (MCR) and the optical metabolic imaging (OMI) index ^[99,102-105]^. Note that NADH also exists in a phosphorylated form (NADPH) that is autofluorescent yet undistinguishable from NADH due to overlapping spectral characteristics. Hence, NADH is typically denoted as NAD(P)H. However, studies have shown that the primary source of autofluorescence is the mitochondrial-bound NADH ^[100,106]^. NADH and NADPH may be separable within live samples based on their lifetime characteristics using FLIM in combination with computational and mathematical modeling ^[107]^. For a detailed description of lifetime image acquisition and analysis, refer to Datta et al, 2020 ^[98]^. Briefly, fluorescence lifetime can be measured by either time-domain FLIM, which measures the time delay between excitation and emission photons, or frequency-domain FLIM, which analyzes the phase and amplitude of fluorescence signals modulated at different frequencies. Time-domain FLIM is the most often used approach and is typically performed using time-correlated single photon counting (TCSPC) electronics. Although frequency-domain FLIM has a faster acquisition speed, time-domain FLIM with TSCPC electronics offers higher accuracy of lifetime estimation and performs better with dim samples and species with long lifetimes. Lifetime images are typically analyzed using the curve fitting ^[99]^ or the fit-free phasor plot ^[108,109]^ methods to extract the lifetime decay values. Curve fitting is applied to time-domain TSCPC FLIM, while phasor analysis can be applied to both types of FLIM measurements. The phasor method has the unique advantage of visually displaying all endogenous fluorophores in a region of interest, not just NAD(P)H or FAD; thereby, one can detect additional species or check for contaminating signals.

FLIM of NAD(P)H and FAD in vitro and in vivo samples are well-documented in various contexts, including tissue engineering, neuroscience, and cancer ^[96,98,110]^. The metabolic imaging of immune cells is more recent. Several studies reported the functional assessment of innate and adaptive immune cells in vitro by two-photon microscopy of NAD(P)H and FAD ^[111-120]^. These groups successfully performed intensity and lifetime imaging in human neutrophils, T and B cells, mouse immune cells including macrophages, neutrophils, dendritic cells, T and B cells, and several cell lines including THP-1 monocytes, RAW264.7 macrophages, and NK-92 cells, demonstrating that it is a valuable tool in immunometabolism. In most cases, TCSPC electronics were used for time-domain lifetime imaging, with one study opting for frequency-domain FLIM ^[111]^, and estimated lifetimes with the curve-fitting and phasor plot methods. Several intravital proof-of-principles studies have shown that FLIM of NAD(P)H and FAD can also be used to assess the intracellular metabolism of immune cells in situ in a living animal ^[121-125]^. These studies used two-photon microscopy combined with TCSPC electronics for lifetime imaging and analyzed lifetime decay using the curve fitting method. The first in vivo study examined macrophages in the context of an intact tumor microenvironment in the PyVT mouse model of human breast cancer ^[121]^. Mammary tumors in whole mice were observed through a surgically implemented Mammary Imaging Window. Macrophages and tumor cells could be distinguished based on differences in metabolite fluorescence intensity and NADH lifetime signatures. Macrophages had high FAD intensity, while tumor cells had high NADH intensity. In addition, FAD^HI^ macrophages also had significantly reduced NAD(P)H mean lifetime compared to tumor cells, suggesting a glycolytic-like metabolism ^[121]^. A study using larval zebrafish models of sterile and infected injuries with different degrees of macrophage inflammation demonstrated that FLIM of NAD(P)H and FAD can distinguish pro-inflammatory and pro-resolving macrophage populations in living animals ^[122]^. This study corroborated its findings by measuring redox metabolites in injured tissues using targeted metabolomics ^[122]^. Interestingly, the pro-inflammatory population exhibited a more oxidized intracellular environment compared to the pro-resolving. Pharmacological treatment of the pro-inflammatory wound with Metformin, an inhibitor of complex I of the electron transport chain system ^[126]^, suggested that mitochondrial ROS may have contributed to the oxidized signature. This is consistent with recent findings in a mouse model of wound healing, which showed that early-phase pro-inflammatory macrophages required mitochondrial ROS production ^[127]^. Immune cells can be successfully imaged not only in living zebrafish but also in live mouse models ^[123-125]^. Lifetime measurements could discern heterogeneity amongst normal tissue-resident macrophages and differentiate between healthy and tumor-associated macrophages in living mice with pancreatic tumors induced by Panc02 pancreatic cancer cells ^[123]^. Tumor-associated macrophages were more oxidized and had lower NAD(P)H and FAD mean lifetimes than macrophages in healthy tissue (ear dermis) ^[123]^. Healthy and tumor-associated T cells could also be distinguished based on lifetime signatures, as demonstrated in a live mouse melanoma model using flank tumors induced by B78 melanoma cells ^[124]^. T cells from melanoma tumors had lower NAD(P)H and FAD mean lifetimes than those from healthy tissue (spleen). Furthermore, T cells could be distinguished from tumor cells within the same tumor, where T cells had higher FAD and lower NAD(P)H mean lifetimes than tumor cells ^[124]^. Another study tracked metabolic changes in T cells within the lymphatic nodes of melanoma-bearing mice over time as the tumor developed ^[125]^. The authors detected an increase in unbound NADH (α_1_) and a decrease in protein-bound NADH (α_2_). This study applied a triple-exponential decay model to calculate the contribution of NADPH (α_3_) that was present only in the large tumor-bearing mice ^[125]^. The changes in the NAD(P)H and FAD lifetime characteristics of tumor-associated T cells detected by these groups indicated a shift toward a glycolytic metabolism, consistent with prior work that identified a glycolytic phenotype in T cells ^[128]^.

In addition to the advantages of having capabilities for live imaging with spatial and temporal resolution and use on human samples, the optical redox ratio and lifetime components (α_1_, α_2_, τ_1_, τ_2_, τ_m_) can be readily used in machine learning classification models and other algorithms to perform subpopulation analysis and classify immune cells based on their subtypes and activation states ^[98,113,115,120,123,129-132]^. For example, logistic regression and random forest models could classify human T cells according to activation state (quiescent or activated) and subtype (CD3^+^CD8^+^ or CD3^+^CD4^+^) with 97% accuracy ^[115]^. However, there are several caveats to keep in mind. First, FAD lifetime is sensitive to the levels of NAD^+^ in the cell, as decreases in the fluorescence lifetime and intensity of protein-bound FAD were observed due to quenching by NAD^+ [133]^. Additionally, other cellular sources of endogenous fluorescence may contribute to NAD(P)H and FAD autofluorescent signals and create a source of error in intensity and lifetime measurements ^[98,134]^. For example, signals collected in the FAD channel may originate from other sources, such as flavin mononucleotide (FMN) and lipofuscin. However, these sources can be distinguished from FAD based on lifetime characteristics ^[98]^. In addition, only a small portion of FAD exists in a free, unbound state. The majority of FAD is bound to enzymes called flavoproteins, such as lipoamide dehydrogenase (LipDH) and electron transfer flavoprotein, which contribute about 75% of the FAD fluorescence; the remaining comes from flavoproteins unrelated to metabolism ^[96,134]^. These caveats underscore that the biological interpretation of the metabolite signatures is not straightforward, as NAD(P)H and FAD play a role in multiple metabolic pathways, and their lifetimes can be affected by numerous enzymes ^[135,136]^. In addition, it is important to remember that metabolic pathways are interconnected ^[137]^. The NAD(P)H and FAD lifetime signatures reflect *global* changes in the cell. It is also difficult to discern changes in specific metabolic pathways that contribute to the lifetime signature of the cells. The changes in the intensity and lifetime measurements and their correlation with the metabolic changes in the cell, such as a shift towards glycolysis or oxidative phosphorylation, are only predicted correlations; actual measurements do not always match the predicted correlations. For example, when the authors treated zebrafish larvae with 2-deoxy-d-glucose, an inhibitor of glycolysis, they measured a reduced optical redox ratio in macrophages of treated larvae compared to control, as expected based on their definition (I_NAD(P)H_/I_NAD(P)H_+I_FAD_), however NAD(P)H τ_m_ decreased instead of increasing ^[122]^. Additional work with metabolic inhibitors or genetic perturbation is required to define the contribution of specific metabolic pathways to the changes in intensity and lifetime signatures, keeping in mind that disturbing one pathway may influence others downstream. These correlations will likely be context-, species- and cell-specific. Another disadvantage is the much-reduced coverage in the number of metabolites that can be simultaneously measured in a sample, in contrast to metabolomics, which detects hundreds of metabolites. To increase coverage, FLIM technologies can be combined with other sources of contrast ^[134]^. For instance, other endogenous fluorophores exist, such as amino acids (eg, phenylalanine, tryptophan, tyrosine), structural proteins (eg, collagen), pigments, and vitamins ^[98,101]^. One can also employ the phasor plot method and detect additional species besides NAD(P)H and FAD in the fluorescence lifetime decay, such as oxidized lipids ^[98,109,138]^.

There are a few considerations when imaging immune cells by FLIM in living animals. First, a contrast source may need to be introduced to perform lifetime measurements in specific cell types in a field of view. This can be achieved by using transgenic animals expressing a fluorescent protein in a cell-specific manner. The spectral properties of the contrast source need to be compatible with those of NAD(P)H and FAD; mCherry has been validated to work well with NAD(P)H and FAD, while GFP can be imaged with NAD(P)H only ^[113,122]^. Second, in typical in vitro measurements of stationary cells, NAD(P)H and FAD are imaged sequentially. However, in living animals, one should consider cell movement during acquisition time; immune cells are highly motile. This may necessitate simultaneous imaging of NAD(P)H, FAD, and the cell-specific contrast source. This can be addressed by using the wavelength mixing approach ^[139]^, where NAD(P)H is excited with 750 nm, mCherry with 1041 nm, and a 2-color excitation with 750 and 1041 nm is used to create a virtual wavelength of 895 nm to excite FAD ^[113,139]^. Since 750 nm is used to excite FAD, NADH is also excited, creating the potential concern for the bleed-through of the tail end of NAD(P)H emission in the FAD image ^[99]^. This can be minimized by using custom-made filters to collect FAD emission signals only, and the phasor plot method can be used to evaluate contamination by other endogenous species in the FAD image. Nevertheless, excitation of FAD using wavelength mixing has been shown to produce similar FLIM signatures as with direct excitation ^[113]^, and has been successfully applied for imaging in vivo ^[122-124]^.

Overall, metabolic imaging by FLIM of NAD(P)H and FAD is still a developing technology but has reached a point where it is technologically ready to be applied in biological studies. The field continues to innovate, driven by the need for diagnostics and mechanistic studies in medicine and basic science. Further advancements in the field will undoubtedly bolster the utility of this technique and allow its routine use in cell biology studies. One necessary advancement is faster acquisition, as it currently takes approximately one minute per image to collect enough photons to fit and estimate the fluorescence decay accurately. Although low temporal resolution is possible already, efforts are ongoing to achieve faster acquisition to enable better capabilities for time-lapse and z-series acquisition ^[77,134,140]^. Alternatively, phasor-based approaches can be used for faster acquisition ^[134,141,142]^. One study imaged microglia in the injured brain of zebrafish and was able to discern differences in the lifetime before and after injury ^[142]^. However, this approach has yet to be thoroughly tested for functional and phenotypic analyses of immune cells. Furthermore, the field would also benefit from clarifying and standardizing NAD(P)H and FAD denotation, image acquisition, and data analysis and interpretation; for example, since several different versions of the optical redox ratio exist, comparing results across different studies is difficult. Lastly, although live imaging is one of the advantages of this technique, it is currently being tested to determine whether FLIM can reliably image NAD(P)H and FAD in fixed samples ^[143-145]^, as this would be highly beneficial in the analysis of clinical samples that are often fixed and stored.

## 4. Conclusions

We are currently experiencing an exciting phase in immunometabolism. In addition to the well-established single-cell approaches ^[4]^, several imaging-based options are available to examine intracellular metabolism within a native context, summarized in Table 2. These single-cell and imaging-based approaches can be used in parallel with the traditional workhorse methods, comprising an arsenal of tools for researchers to study immunometabolism in situ across a broad range of complex biological processes, including infection, wound healing, and cancer. Although mammalian models are the preferred choice, zebrafish (*Danio rerio*) have emerged as a valuable vertebrate system and have been steadily gaining popularity in immunological studies over the past two decades. The sequencing and annotation of the zebrafish genome revealed that 70% of human genes have at least one zebrafish orthologue ^[146]^, making them useful in modeling human diseases ^[147]^. Zebrafish have the distinct advantage of being readily amenable to most imaging modalities due to their optical transparency during larval stages of development ^[148]^. Additionally, there are zebrafish mutants available that stay transparent into adulthood ^[149]^. *Danionella cerebrum* is another emerging fish model that naturally stays transparent throughout life ^[150]^. For this reason, biosensors for single-cell imaging are more likely to be successfully implemented in these organisms. By leveraging high spatial and temporal resolution and sensitivity, single-cell Raman microscopy and FLIM may enable the identification of metabolic signatures associated with specific immune cell types and activation states while also uncovering phenotypic heterogeneity within cell populations. However, given the high cost and the required expertise in instrumentation and data analysis, the ability of a single laboratory to implement these technologies will vary, and they may be more accessible via a core facility with in-house expertise or collaboration with other laboratories. Spatial metabolomics and imaging MS are also expensive and highly multidisciplinary technologies, requiring expertise in instrumentation, sample preparation, data acquisition, and analysis. These factors make their implementation by a single laboratory also difficult and are more accessible through a core facility or collaboration. Implementation of enzyme histochemistry and biosensor imaging is achievable by a single laboratory, although biosensor imaging also requires a relatively sophisticated microscope setup (such as a confocal spinning disk microscope and high-performance cameras) and expertise in data acquisition and analysis. The ability to provide spatially and temporally resolved metabolic profiles will offer a more comprehensive understanding of the spatial organization of metabolic pathways within immune cells. As these technologies continue to advance, they promise to contribute to our understanding of metabolic regulation of immune cell function and pave the way for better diagnostics and targeted therapeutic interventions in immune-related disorders.

## Figures and Tables

**Table 1.** A partial list of fluorescent probes and biosensors available for metabolic analysis in animals.

Sensor	Target	References	Examples in living animals
BODIPY FL C_16_	Fatty acid	[28]	[29-31]
2-NBDG	Glucose	[32,33]	[34,35]
Red Glifons	Glucose	[36]	[36]
Mito Timer	Mitochondrial health	[37]	[37-39]
Citron1	Citrate	[40,41]	None
BioITA	Itaconate	[42]	None
Frex	NADH	[43]	None
FiNad	NAD^+^	[44]	[44]
SoNar	NAD^+^/NADH	[45]	[46]
mt-SoNar	Mitochondrial NAD^+^/NADH	[47]	None
iNap	NADPH	[48]	[48]
NADPsor	NADP^+^	[49]	None
Shikimate	NADP^+^/NADPH	[50]	None
ATeams	ATP	[51]	[52]
GO-ATeam	ATP	[53]	[54,55]
PercevalHR	ATP/ADP	[56]	[57]
Hyper7	H_2_O_2_	[58]	[58]
roGFP2	Thiol/disulfide equilibrium	[59,60]	[61-64]
roGFP-Orp1	H_2_O_2_	[65]	[52,66-68]
Grx1-roGFP2	GSH/GSSG	[69]	[67,70-73]
NERNST	NADP(H) redox states via oxidoreduction of the roGFP2	[74]	[74]

**Table 2.** Summary of techniques available for in situ metabolic analysis.

Features	Spatial metabolomics, Imaging MS	Enzyme histochemistry	Fluorescent probes and biosensors	Raman microscopy	FLIM
Specialized instrumentation	✓			✓	✓
Complex data analysis	✓		✓	✓	✓
Big data	✓		✓	✓	✓
Broad detection of biomolecules in one sample (~100)	✓				
Ability to capture heterogeneity	✓	✓	✓	✓	✓
Straightforward biological interpretation	✓	✓	✓		
Spatial resolution	✓	✓	✓	✓	✓
Temporal resolution			✓	✓	✓
Living animals			✓	✓	✓
Label-free				✓	✓
Types of biomolecules Detected	metabolites, proteins, lipids, drugs	dehydrogenases	amino acids, fatty acids, carbohydrates, metabolites	amino acids, nucleic acids, lipids, carbohydrates, metabolites	NAD(P)H, FAD, other endogenous fluorophores
Likelihood of implementation by a single laboratory	low	high	medium	low	low

## Abbreviations:

BCARS: broadband coherent anti-Stokes Raman scattering
CRM: confocal Raman microspectroscopy
cRSI: confocal Raman spectroscopic imaging
FAD: flavin adenine dinucleotide
Fh1: Fumarate hydratase
FLIM: fluorescence lifetime imaging microscopy
MALDI: matrix-assisted laser desorption ionization
MS: mass spectroscopy
NADH: nicotinamide adenine dinucleotide
NADPH: nicotinamide adenine dinucleotide phosphate
SRS: stimulated Raman spectroscopy
